# Fruit Quality Characteristics of Service Tree (*Sorbus domestica* L.) Genotypes

**DOI:** 10.1021/acsomega.3c01788

**Published:** 2023-05-23

**Authors:** Akgul Tas, Muttalip Gundogdu, Sezai Ercisli, Erdal Orman, Kenan Celik, Romina Alina Marc, Martina Buckova, Anna Adamkova, Jiri Mlcek

**Affiliations:** † Department of Plant and Animal Production, Seben İzzet Baysal Vocational School, Bolu Abant Izzet Baysal University, 14750 Seben Bolu, Turkey; ‡ Department of Horticulture, Faculty of Agriculture, Bolu Abant Izzet Baysal University, 14030 Bolu, Turkey; § Department of Horticulture, Faculty of Agriculture, Ataturk University, 25240 Erzurum, Turkey; ∥ HGF Agro, Ata Teknokent, TR-25240 Erzurum, Turkey; ⊥ Ataturk Horticultural Central Research Institute, 77100 Yalova, Turkey; # GAP International Agricultural Research and Training Center, 21000 Diyarbakır, Turkey; ¶ Food Engineering Department, Faculty of Food Science and Technology, University of Agricultural Sciences and Veterinary Medicine, 400372 Cluj-Napoca, Romania; ∇ Technological Transfer Center “CTT-BioTech”, University of Agricultural Sciences and Veterinary Medicine Cluj-Napoca, Calea Floreşti Street, No. 64, 400509 Cluj-Napoca, Romania; ○ Department of Food Analysis and Chemistry, Faculty of Technology, Tomas Bata University in Zlin, Vavreckova 5669, 76001 Zlin, Czech Republic

## Abstract

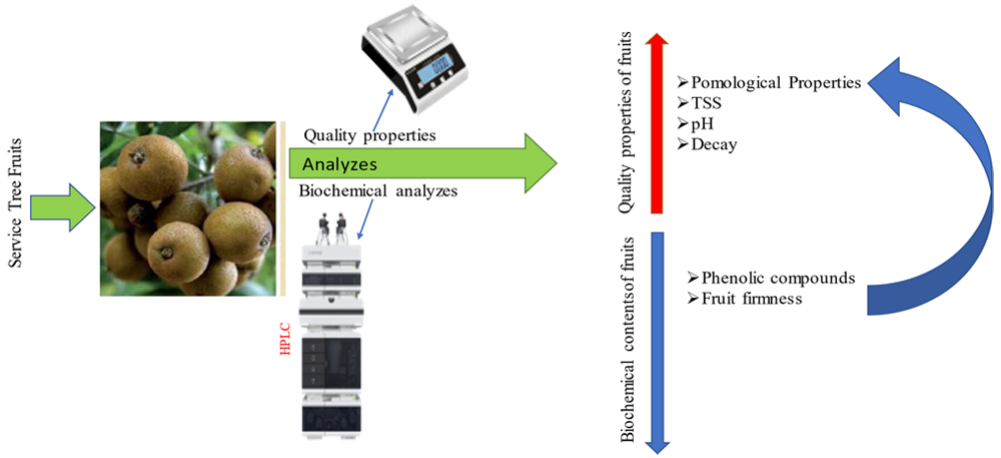

In this study, agro-morphological properties, phenolic compounds, and organic acid contents in the fruits of service tree (*Sorbus domestica* L.) genotypes naturally grown in Türkiye (Bolu) were determined. The fruit weights of genotypes were found to be quite variable, ranging from 5.42 g (14MR05) to 12.54 g (14MR07). The highest L*, a*, and b* fruit external color values were found to be 34.65 (14MR04), 10.48 (14MR09), and 9.10 (14MR08), respectively. The highest chroma and hue values were recorded as 12.87 (14MR09) and 49.07 (14MR04), respectively. 14MR03 and 14MR08 genotypes exhibited the highest amount of soluble solid content and titratable acidity (TA) as 20.58 and 1.55%, respectively. The pH value was found to be in the range of 3.98 (14MR010)–4.32 (14MR04). Chlorogenic acid (14MR10, 48.49 mg/100 g), ferulic acid (14MR10, 36.93 mg/100 g), and rutin (14MR05, 36.95 mg/100 g) were predominant phenolic acids observed in the fruits of service tree genotypes. The predominant organic acid in all fruit samples was malic acid (14MR07, 34.14 g/kg fresh weight basis), and the highest quantity of vitamin C was detected at 95.83 mg/100 g in genotype 14MR02. Principal component analyses (%) were performed to determine the correlation between the morphological–physicochemical (60.6%) and biochemical characteristics of genotypes (phenolic compounds: 54.3%; organic acids and vitamin C: 79.9%). It was determined that measured genotypes were important genetic resources in terms of nutritional value.

## Introduction

*Sorbus* genus, which belongs to the family Rosaceae (*Rosaceae*) of the order Rosales, sheds its leaves in winter in certain periods, is pollinated by insects that are highly resistant to hot conditions, and grows quickly up to 3–25 m in height.^1^ The genus is generally of economic importance as an ornamental plant and includes about 100 species. 12 out of 100 species of this genus are known to exist in Türkiye. The most important species found in Türkiye are maple-leaved service trees (*Sorbus torminalis* Linnaeus), bird ash (*Sorbus aucuparia* L.), and service trees (*Sorbus domestica* L.). Service trees, which can be quite tolerant of many different soil types, including stony and calcareous soils, can be found on south-facing slopes with mild climate conditions in extremely dry, hot, and nutrient-poor areas where the Mediterranean climate is observed.^2^ Service tree is mostly found in southern and central Europe, mainly in the Balkan peninsula, Italy, and southern France, but it can occasionally grow naturally in North Africa and the Caucasus.^1^ In Türkiye, it is mostly found in the Marmara Region, the north of Central Anatolia, the Central and Western Black Sea Region, the inner part of the Aegean, the Lakes region, and the Hatay region. Its consumption is quite common, especially in provinces in the gateway regions (such as Tokat, Amasya, and Kastamonu).^3^ Service trees, which can bloom in May and June, are trees that can bear fruit from July to the end of October. Although the blossoms are white in color, the trees, which can rarely be pink, have male and female organs together in the same flower. Its leaves are about 1 cm wide and 3–6 cm long, with toothed edges.^4,5^

As in many other countries, service tree has also become popular in Türkiye thanks to its benefits on human health.^6−8^ Service tree is important as an ornamental plant whose fruits in particular can contribute to human health.^9^ One of the important components in the fruits is phenolic substances. This substance is effective in metabolic processes in fruits. Phenolic substances contribute to the formation of taste and flavor in fruits, especially in leaving a bitter taste in the mouth. Anthocyanins, which are also included in phenolic compounds, affect the consumer with different color formations they create.^10−14^ Service tree, whose fruits are known to be important in the field of medicine, can be evaluated in the category of ’medicinal plants’ thanks to its anti-inflammatory, antioxidant, and many other beneficial properties.^14^ It is known that the consumption of service tree fruits has a positive effect on the prevention of many diseases in humans, such as diabetes, liver disease, winter diseases, and spring fatigue. Due to the positive effects of this fruit on the intestinal flora ofbeneficial microorganisms, it has become a functional food that many people prefer.^7^

In this study, morphological, physicochemical, and biochemical characteristics of fruits belonging to service tree genotypes were determined. As a result of the analysis, statistical distributions and definitions of service tree genotypes were made in terms of morphological and biochemical characteristics.

## Materials and Methods

### Fruit Material

In this research, some service tree genotypes grown in fields of Türkiye, namely Bolu province, were determined. Fruit samples taken from service tree genotypes were placed in appropriate containers, labeled, and transported to the laboratory. After the agro-morphological characteristics of these fruit samples were determined, they were stored at −80 °C for biochemical analysis. Morphological–physicochemical analyses were carried out at the Bolu Abant İzzet Baysal University Faculty of Agriculture Laboratory. Among the biochemical analyses, phenolic compounds, organic acids, and vitamin C analyses were carried out at the Bolu Abant İzzet Baysal University Scientific, Industrial, and Technological Application and Research Center.

### Determination of Agro-Morphological Characteristics of Fruits

Fruit weight was obtained by randomly taking 20 fruits from each genotype and weighing them individually on a scale sensitive to 0.01 g. After taking the arithmetic average of the obtained values, the fruit weight (g) values of the genotypes were determined separately. The width and length of the fruit and core were measured separately, with a caliper sensitive to 0.01 mm of 10 fruit samples taken randomly from each genotype. Fruit width was measured as the widest part in the equatorial region, and fruit length was measured as the longest part between the fruit stalk and the flower tip. After taking the arithmetic average of the obtained values, the fruit and core width and height values of the genotypes were determined separately in mm.^15−17^

Fruit taste was determined by a degustation group of five people using the sensory scale of bad, moderate, good, and very good. Taste was scored as poor 1, moderate 2, good 3, and very good 4. Fruit taste was determined after arithmetic averages were taken by adding the points to be given.^18^ Fruit astringency was determined by tasting the fruit samples and taking the average of the sensory scores given out of 1–4 after adding them. In the evaluation, the fruit is expressed as not acrid 1, slightly acrid 2, moderately acrid 3, and acrid 4 in terms of astringency.^19^ Fruit aroma was determined according to the aroma values of service tree fruit genotypes as 1: good, 2: medium, and 3: bad.^20^

Fruit skin color was measured in terms of L*, a*, and b* with a Konica Minolta CR-400 brand colorimeter. L* is the luminance value, 0 indicates black, and 100 indicates white. Accordingly, a* indicates red, −a* green, b* yellow, and −b* blue. Color values for each fruit were calculated by means of three measurements taken reciprocally in the equatorial region of the fruit.^21^ The soluble solid content (SSC) was determined as % by a hand refractometer (Atago PAL-1, Washington, USA) in the juice obtained by squeezing.^22^

In order to determine the pH (Thermo, OrionStar A111, ABD) of the fruit in terms of fruit juice pH, a homogeneous juice mixture was obtained by squeezing the juice of 20 randomly selected fruits. The measurement was made when the temperature of the juice was at room temperature. 10 mL of this juice mixture was taken into a 50 mL beaker, and the electrode of the pH meter was immersed in the juice mixture. After waiting until the value stabilized, the value read was recorded as the pH value.^23^

In order to determine the titratable acidity (TA) content of the fruits examined in terms of TA in fruit juice, 20 fruits from each genotype were squeezed in a cheesecloth to extract their juices. 10 mL of fruit juice obtained from the fruit juice in this way was diluted to 50 mL with distilled water. These diluted samples were titrated with 0.1 N NaOH until pH 8.1. The acid value in terms of malic acid according to the amount of spent NaOH was calculated according to the formula below.^22^ The SSC/TA ratio was obtained by dividing the SSC value by the TA value.^24^
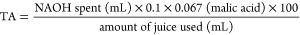


### Analysis of Biochemical Properties of Fruits

#### Analysis of Phenolic Compounds

In this study, phenolic compounds were determined. In the separation of phenolic compounds by HPLC, a modified method of Rodríguez-Delgado et al.^25^ was used. 50 g of sample was diluted 1:1 with distilled water and centrifuged at 15,000 rpm for 15 min. Then, the upper part was filtered with 0.45 μm Millipore filters and injected into HPLC. Chromatographic separation was performed on an Agilent 1100 (Agilent) HPLC system using a DAD detector (Agilent, USA) and a 250 × 4.6 mm, 4 μm ODS column (HiChrom, USA). Solvent A, methanol–acetic acid–water (10:2:88), and solvent B, methanol–acetic acid–water (90:2:8), were used as mobile phases. Separation was performed at 254 and 280 nm; the flow rate was determined as 1 mL/min, and the injection volume was determined as 20 μL.

#### Analysis of Organic Acids

The samples were kept in a deep freezer (−80 °C) until the analysis. In the study, citric acid, tartaric acid, malic acid, succinic acid, and fumaric acid contents of organic acids were determined in a service tree fruit. The method given by Bevilacqua and Califano^26^ was modified and used for the extraction of organic acids. 50 g of the service tree samples were taken and transferred to centrifuge tubes. 20 mL of 0.009 N H_2_SO_4_ was added to these samples and homogenized (Heidolph Silent Crusher M, Germany). Then, it was mixed for 1 h on a shaker (Heidolph Unimax 1010, Germany) and centrifuged at 15,000 rpm for 15 min. The aqueous fraction separated in the centrifuge was first passed through a coarse filter paper, then twice through a 0.45 μm membrane filter (Millipore Millex-HV Hydrophilic PVDF, Millipore, USA), and finally through the SEP-PAK C18 cartridge. Samples were analyzed in an HPLC instrument (Agilent HPLC 1100 series G 1322 A, Germany). An Aminex HPX-87 H, 300 mm × 7.8 mm column (Bio-Rad Laboratories, Richmond, CA, USA) was used in the HPLC system, and the device was controlled by a computer with an Agilent package program. The DAD detector (Agilent, USA) in the system is tuned to 214 and 280 nm wavelengths. In the study, 0.009 N H_2_SO_4_ passed through a 0.45 μm membrane filter was used as the mobile phase.

#### Analysis of Vitamin C

50 g of fresh service tree fruit was homogenized (Heidolph Silent Crusher M, Germany); then, 5 g of serving fruit sample was taken from this sample and transferred to the test tube. 5 mL of 2.5% M-phosphoric acid solution was added to this sample. The mixture was centrifuged at 6500*g* for 10 min at +4 °C. 0.5 mL was taken from the clear part in the centrifuge tube and made up to 10 mL with 2.5% M-phosphoric solution. This mixture was filtered through a 0.45 μm Teflon filter and injected into the HPLC device. In HPLC analysis, vitamin C (ascorbic acid) was carried out on a C18 column (Phenomenex Luna C18, 250 × 4.60 mm, 5 μm). The column furnace temperature was set at 25 °C. Ultrapure water with the pH level adjusted to 2.2 with H_2_SO_4_ at a flow rate of 1 mL/min was used as the mobile phase in the system. Readings were made in a DAD detector at a wavelength of 254 nm. l-ascorbic acid (Sigma A5960) prepared at different concentrations (50, 100, 500, 1000, and 2000 ppm) was used to identify and quantify the vitamin C peak.^20^

#### Statistical Analysis

Student’s *t* (LSD)-test was used to compare the means in the evaluation of agro-morphological and biochemical data. Statistical analyses were performed with the Statistical Package for the Social Sciences (SPSS) statistical program. In this study, principal component analysis (PCA) was performed by the “ggbiplot”^27^ package program to determine the relationships of genotypes with morphological and physicochemical characteristics.

## Results and Discussion

### Morphological–Physicochemical Properties of Fruits

Türkiye is a country rich in flora due to its geographical location and has a wide range of fruit species. Service tree fruit species is one of the prominent species in recent years due to the important phytochemicals it contains in the composition of its fruits. In this study, service tree fruit samples of the selected service tree genotypes were taken, and the necessary measurements and analyses were made in order to determine the morphological and physicochemical properties of the fruits. Accordingly, statistical differences were determined between physicochemical properties and genotypes (*p* ≤ 0.05). In this study, statistically significant differences were found between genotypes in terms of fruit weight (*p* ≤ 0.05). Among the examined genotypes, the highest fruit weight was detected in 14MR07 genotype (12.54 g) and the lowest fruit weight in 14MR05 genotype (5.42 g). 14MR07 genotype was followed by 14MR06 (11.37 g), 14MR04 (10.92 g) and 14MR01 (10.48 g) genotypes, respectively. Accordingly, 14MR07, 14MR06, 14MR04, and 14MR01 genotypes were determined as prominent genotypes in terms of high fruit weight (Table 1). In a study on service trees, the highest fruit weight was found to be 9.8 g.^28^ In a study in Slovakia in which the morphological characteristics of 18 different service tree genotypes were determined, fruit weight ranged from 3 to 21.8 g.^29^ In a study investigating fruit weight in 167 different service tree genotypes in Slovakia, the highest fruit weight was found to be 18.64 g.^30^ In a study in which the performance of service tree genotypes was determined in Tokat (in Türkiye), fruit weight ranged from 5.58 to 12.28 g.^31^ In another study conducted on service tree fruits in Tokat province, the highest fruit weight was found to be 18.23 g.^32^ In a study on service tree fruits, the highest fruit weight was found to be 15.48 g.^33^ In a study in which fruit weight was determined in service tree fruit, fruit weight was determined as 5.30 g.^34^ In another study, fruit weight values ranged from 6.9 to 16 g.^35^ In a study conducted on service tree fruits in Kocaeli (in Türkiye) province, the highest fruit weight was found to be 7.28 g.^36^ In the studies carried out by different researchers, different results can be obtained due to the differences in cultural practices and the geographical conditions in which the fruits are grown. These literature results regarding service tree fruit weights were similar to the fruit weight findings obtained in this study.

**Table 1:** Fruit Weight, Fruit Width, Fruit Length, Core Weight, Core Width, and Core Length Characteristics of Fruits Belonging to Service Tree Genotypes

genotypes	fruit weight (g)	fruit width (mm)	fruit length (mm)	core width (mm)	core length (mm)	fruit stalk length (mm)	fruit stalk thickness (mm)
14MR01	10.48 ± 0.4a–d^a^	25.2 ± 0.3abc	26.50 ± 0.6abc	5.06 ± 0.1b–e	6.78 ± 0.1def	11.69 ± 0.6b	1.25 ± 0.09bc
14MR02	7.19 ± 0.3ef	21.69 ± 0.5d	24.94 ± 0.5c	4.49 ± 0.1e	6.43 ± 0.1f	11.49 ± 1.8b	1.10 ± 0.09c
14MR03	9.26 ± 0.2b–e	24.42 ± 0.2bc	25.38 ± 0.3bc	4.36 ± 0.2e	7.4 ± 0.1cd	13.88 ± 1.1b	1.10 ± 0.04c
14MR04	10.92 ± 0.4abc	26.37 ± 0.4ab	25.00 ± 0.4bc	5.37 ± 0.2abc	6.72 ± 0.0def	6.10 ± 0.9b	1.48 ± 0.16abc
14MR05	5.42 ± 0.2f	20.74 ± 0.3d	20.89 ± 0.3d	4.58 ± 0.2de	6.24 ± 0.1f	13.25 ± 1.9b	1.31 ± 0.14bc
14MR06	11.37 ± 0.9ab	25.90 ± 0.7abc	27.39 ± 0.6ab	5.51 ± 0.2ab	8.13 ± 0.1ab	31.30 ± 2.8a	2.00 ± 0.05a
14MR07	12.54 ± 0.7a	27.21 ± 0.6a	28.73 ± 0.7a	5.25 ± 0.1bcd	8.42 ± 0.1a	31.01 ± 1.3a	1.65 ± 0.04abc
14MR08	8.90 ± 0.3cde	24.78 ± 0.2bc	26.03 ± 0.5bc	5.33 ± 0.1abc	7.53 ± 0.2bc	24.34 ± 1.4ab	1.87 ± 0.22a
14MR09	9.38 ± 0.3b–e	24.66 ± 0.3bc	25.86 ± 0.4bc	4.73 ± 0.1cde	7.29 ± 0.1cde	14.20 ± 0.6b	1.77 ± 0.21ab
14MR10	8.74 ± 0.4de	24.07 ± 0.3c	25.76 ± 0.3bc	6.01 ± 0.1a	6.66 ± 0.1ef	12.12 ± 1.1b	1.32 ± 0.05bc

a The difference between the means with the same letter in the same column is significant at the *p* < 0.05 level.

When the data were examined in terms of fruit sizes, the differences between genotypes in terms of fruit width and fruit length were found to be statistically significant (*p* ≤ 0.05). While the highest fruit width was obtained from the 14MR07 genotype with 27.21 mm, the highest fruit length was recorded as 28.73 mm in the same genotype. When we look at the lowest fruit sizes, it was determined that the 14MR05 genotype had both the shortest fruit width (20.74 mm) and the shortest fruit length (20.89 mm). In terms of fruit width, 14MR07 genotype was followed by 14MR04 (26.37 mm), 14MR06 (25.9 mm), and 14MR01 (25.21 mm) genotypes, respectively. 14MR07 genotype was followed by 14MR06 (27.39 mm) and 14MR01 (26.5 mm) genotypes in terms of fruit length, respectively (Table 1). In a study on service trees, fruit length was determined to be between 18.6 and 33.4 mm.^28^ In a study conducted on 18 different service tree genotypes in Slovakia, fruit widths ranged from 16 to 33 mm. In the same study, fruit size was found to be between 18 and 38 mm.^29^ In a study in which fruit size was determined in a service tree fruit variety called Dura, the highest fruit size was found to be 30.25 mm.^37^ In a study in which sizes were determined in the service tree fruit, the highest fruit length was 78 mm and the highest fruit width was 26 mm.^35^ In a study in which the size of service tree fruits was determined in Kocaeli province, the highest fruit length was found to be 17.1 mm and the highest fruit width to be 19.9 mm.^36^ In a study conducted on service tree fruits in Tokat province, fruit length was determined as 29.25 mm and fruit width as 29.99 mm.^32^ Piagnania et al.,^35^ the results of the above-mentioned literature on service tree fruit sizes were similar to the fruit size findings obtained in this study, except for the determination made by fruit size. On the other hand, it is thought that the difference in fruit size may be caused by genotype, geographical location, ecological factors, soil characteristics, and years. When the data were analyzed in terms of core weight, it was seen that the values were not statistically significant (*p* > 0.05).

When the core weight measurements were examined, it was determined that the 14MR01 genotype had the highest value with 0.45 g, while the 14MR02 genotype had the lowest value with 0.26 g (Table 1). In a study conducted on service tree fruits in Tokat province, the core weight was determined as 0.17 g.^32^ According to the literature result determined by the researchers regarding the service tree core weight, the amount of core weight obtained in this study was observed to be higher. When the data were examined in terms of core width, the differences between genotypes in terms of core width were found to be statistically significant at the *p* ≤ 0.05 level. The highest core width was determined as 6.01 mm in 14MR010 genotype, while the lowest core width was recorded in the 14MR03 genotype as 4.36 mm. It was determined that the 14MR010 genotype was followed by the 14MR06 (5.51 mm), 14MR04 (5.37 mm), and 14MR08 (5.33 mm) genotypes, respectively (Table 1). It is thought that these results related to core width in this study may be beneficial in the formation of the literature on service tree fruit.

When the data were examined in terms of core size, the difference between genotypes in the 10 service tree genotypes determined in the study was found to be statistically significant at the *p* ≤ 0.05 level. The highest value in terms of core length was determined as the 14MR07 (8.42 mm) genotype, while the lowest value was measured as the 14MR05 (6.24 mm) genotype. 14MR07 genotype was followed by the 14MR06 (8.13 mm) genotype (Table 2). It is thought that these results determined in this study regarding the core size may be beneficial in the formation of the literature on service tree fruits.

**Table 2:** Fruit Juice pH, Total Amount of SSC, TA, and SSC/TA Ratio Characteristics of Fruits Belonging to Service Tree Genotypes

genotypes	pH	SSC (%)	TA (%)	SSC/TA
14MR01	4.27 ± 0.04a^a^	20.25 ± 1.23ab	0.94 ± 0.08bc	21.00 ± 0.58ab
14MR02	4.28 ± 0.02a	18.83 ± 0.38abc	0.71 ± 0.06c	26.67 ± 2.60a
14MR03	4.21 ± 0.02a	20.58 ± 0.98a	0.95 ± 0.01bc	21.33 ± 0.88ab
14MR04	4.32 ± 0.02a	20.20 ± 0.50ab	1.24 ± 0.09ab	16.00 ± 1.53bc
14MR05	4.14 ± 0.02ab	17.40 ± 0.42abc	1.10 ± 0.09b	15.67 ± 1.45bc
14MR06	4.25 ± 0.05a	17.50 ± 0.51abc	1.26 ± 0.09ab	13.67 ± 1.45c
14MR07	4.16 ± 0.04a	16.63 ± 0.61c	1.26 ± 0.03ab	12.67 ± 0.33c
14MR08	4.27 ± 0.05a	16.47 ± 0.41c	1.55 ± 0.05a	10.33 ± 0.67c
14MR09	4.23 ± 0.03a	17.13 ± 0.35bc	1.09 ± 0.08b	15.33 ± 1.45bc
14MR10	3.98 ± 0.05b	11.17 ± 0.38d	1.12 ± 0.08b	9.67 ± 0.88c

a The difference between the means with the same letter in the same column is significant at the *p* < 0.05 level.

Stem length and stem thickness affect the harvesting process of the fruits as well as the separation and damage of the fruits from the branch. Therefore, these properties of fruits also indirectly affect the storage period. When the data were examined in terms of fruit stem length, the differences between genotypes in terms of fruit stem length were found to be statistically significant at the *p* ≤ 0.05 level. While the highest fruit stem length was determined as 31.3 mm in the 14MR06 genotype, the lowest fruit stem length was recorded in the 14MR04 genotype at 6.1 mm. It was determined that the 14MR06 genotype was followed by the 14MR07 (31.01 mm) and 14MR08 (24.34 mm) genotypes, respectively (Table 2). In a study on service trees, fruit stem length was determined to be between 1.8 and 3.7 mm.^28^ According to this literature result determined by the researchers regarding service tree fruit stem length, the amount of fruit stem length obtained in this study was observed to be higher. When the data were examined in terms of fruit stem thickness, the differences between genotypes in terms of fruit stem thickness were found to be statistically significant at the *p* ≤ 0.05 level. The highest fruit stalk thickness was determined in the 14MR06 genotype at 2 mm, while the lowest fruit stalk thickness was recorded in 14MR02 and 14MR03 genotypes with a value of 1.1 mm. It was determined that the 14MR06 genotype was followed by the 14MR08 (1.87 mm) and 14MR09 (1.77 mm) genotypes, respectively (Table 1). It is thought that these results related to fruit stem thickness in this study may be beneficial in the formation of the literature on service tree fruits. Different researchers may obtain different results due to the geographical conditions in which the fruits are grown and the differences in cultural practices. When the data were examined in terms of fruit juice pH, the pH amounts of the fruit juice belonging to the service tree genotypes were examined, and statistically significant differences were found to be *p* ≤ 0.05. The pH ranges of all genotypes were found to be close to each other (between 4.14 and 4.32), except for the 14MR010 genotype, where the pH value among the genotypes was observed to be the lowest (3.98) (Table 2). In a study measuring the pH in service tree fruit juices, the pH was found to be 4.6.^38^ In a study that measured the pH in service tree fruit juices in Kocaeli, the pH was found to be 4.0.^36^ In a study conducted on service tree fruits in Tokat province, the pH amount was recorded as 3.2.^32^ The above literature results regarding the pH amount of service tree fruit juices were similar to the results of the fruit juice pH amount obtained in this study. When the data were analyzed in terms of total SSC, it was seen that the differences between the service tree genotypes in the amount of SSC were statistically significant at the *p* ≤ 0.05 level. Among the genotypes, the highest amount of SSC was obtained from the 14MR03 genotype with 20.58%, and this genotype was followed by the 14MR01 with a value of 20.25% and the 14MR04 genotype with a value of 20.20%. The least amount of SSC was determined as the 14MR010 genotype with 11.17% (Table 2). In a study on service trees, it was determined that the amount of SSC was between 15.7 and 22.5%.^28^ In a study measuring the amount of SSC in service tree fruits, the highest amount of SSC was found to be 13.07%.^38^ In a study in which the performance of service tree genotypes was determined in Tokat, it was determined that the amount of SSC varied between 30.1 and 41.48%.^31^ In a study conducted in service tree fruits, the amount of SSC was found to be between 19.3 and 31.8%.^35^ In a study conducted on service tree fruits in Kocaeli province, the highest amount of SSC was determined as 20.2%.^36^ In a study conducted on service tree fruits in Tokat province, the highest amount of SSC was found to be 17.73%.^32^ Except for the determination made by Öz Atasever et al.^31^ regarding SSC, the literature results mentioned above regarding the SSC were similar to the findings of the SSC obtained in this study. On the other hand, it is thought that the difference in SSC may be caused by genotype, geographical location, ecological factors, soil characteristics, and years. When the data in terms of TA in fruit juice were examined, statistically significant differences were found when the TA values of the fruit juices belonging to the genotypes were examined (*p* ≤ 0.05). The 14MR08 genotype (1.55%) had the highest TA value among the genotypes. This genotype was followed by the 14MR06 and 14MR07 genotypes with a TA value of 1.26% and the 14MR04 genotype with a TA value of 1.24%, respectively. The lowest (0.71%) TA value was recorded in the 14MR02 genotype (Table 2). In a study investigating TA values in service tree fruits, acidity values ranged from 0.64 to 0.74%.^39^ In a study conducted in service tree fruits, TA values were found between 5.9 and 12.2%.^35^ In a study conducted on service tree fruits in Kocaeli province, the highest TA value was found to be 0.42%.^36^ In a study conducted on service tree fruits in Tokat province, the highest TA value was found to be 10.08%.^32^ In terms of TA values in service tree fruits, when the results of the literature determined above were compared with the results of this study, it was observed that the acidity values determined in this study were partially higher or lower than the results of the examined literature. Accordingly, it is thought that these differences in acidity values may be caused by genotype, geographical location, ecological factors, soil characteristics, and years. When the data were examined in terms of the SSC/TA ratio, the ratio of SSC/TA of fruits belonging to service tree genotypes was examined statistically and found to be significant (*p* ≤ 0.05). According to the results of the analysis, the genotype with the highest SSC/TA ratio (26.67) was determined as 14MR02. The 14MR02 genotype was followed by 14MR03 (21.33) and 14MR01 (21.00) genotypes, respectively. The lowest (9.67) value was determined as the 14MR010 genotype (Table 2). It is thought that these results determined in this study regarding the SSC/TA ratio may be beneficial in the formation of the literature on service tree fruits.

The most widely used and most popular color measurement method is the L*a*b* method. In this color range, L* indicates lightness/darkness, and a* and b* are chromaticity coordinates. It is red in the +a* direction, green in the −a* direction, yellow in the +b* direction, and blue in the −b* direction. The center is achromatic. As a* and b* values increase and move away from the center, the vividness of the color also increases.^21^ Accordingly, when the data were examined in terms of the fruit skin color, the differences in terms of the L* value in service tree genotypes were found to be statistically significant (*p* ≤ 0.05). The change of L* value from 0 to 100 indicates that service tree fruits are darker (0) or lighter (100) in color. According to the measurements, the lightest fruit color among the genotypes was detected in the 14MR04 genotype with a L* value of 34.65. This genotype was followed by 14MR08 (33.97) and 14MR05 (33.91) genotypes, respectively. 14MR07 genotype had the darkest fruit color with a 30.05 L* value (Table 3). In a study in which the skin color of service tree fruit was measured, the L* value, which is an important parameter, was found to be between 33.3 and 56.3 values.^35^ In a study in which the skin color of service tree fruits was measured in the province of Tokat, the highest L* value was found to be 26.76.^32^ The above-mentioned literature results regarding the L* value, which is an important criterion in measuring the color of the service tree fruit peel, showed similarities with the L* value findings obtained in this study.

**Table 3:** *L*, a*, b*, Chroma, and Hue Characteristics Used in the Measurement of the Skin Color of Fruits Belonging to Service Tree Genotypes

genotypes	L*	a*	b*	chroma	hue
14MR01	32.41 ± 1.26^NS^	7.14 ± 0.29ab	7.14 ± 0.35^NS^	11.30 ± 1.57^NS^	44.98 ± 0.75a–d^a^
14MR02	31.23 ± 0.51	7.17 ± 0.93ab	8.45 ± 0.63	11.63 ± 1.00	46.98 ± 1.34ab
14MR03	31.08 ± 0.58	7.50 ± 0.33ab	5.04 ± 0.73	9.08 ± 0.66	33.18 ± 2.69cd
14MR04	34.65 ± 1.16	6.53 ± 0.63b	7.62 ± 0.97	10.05 ± 1.13	49.07 ± 1.35a
14MR05	33.91 ± 1.81	7.72 ± 0.97ab	7.27 ± 0.99	10.89 ± 0.61	43.01 ± 6.70a–d
14MR06	30.49 ± 0.64	7.89 ± 0.39ab	6.54 ± 0.75	10.31 ± 0.62	39.18 ± 3.15a–d
14MR07	30.05 ± 0.75	8.64 ± 0.82ab	5.41 ± 0.99	9.80 ± 1.35	31.24 ± 2.47d
14MR08	33.97 ± 1.32	8.21 ± 0.97ab	9.10 ± 1.10	12.29 ± 1.38	47.76 ± 2.46ab
14MR09	31.24 ± 1.03	10.48 ± 0.67a	7.37 ± 1.10	12.87 ± 1.13	34.05 ± 2.98b–d
14MR10	32.57 ± 0.43	8.83 ± 0.39ab	8.40 ± 0.55	12.25 ± 0.62	42.99 ± 1.31a–c

a The difference between the means with the same letter in the same column is significant at the *p* < 0.05 level. NS: Non significant.

Differences in a* values in color measurements of service tree genotypes were found to be statistically significant (*p* ≤ 0.05). According to the fruit color measurements, +*a* value shows a red color and −*a* value shows a green color. Among the service tree genotypes, 14MR09 genotype had the highest a* value of 10.48, followed by 14MR010 genotype with a 8.83 a* value and 14MR07 genotype with a 8.64 a* value, respectively. In addition, the 14MR04 genotype had the lowest a* value with 6.53 (Table 3). In a study conducted to measure the fruit skin color of service tree fruits, the a* value, which is the criterion, was found to be between 3.54 and 8.92.^38^ In a study in which the skin color of service tree fruits was measured in the province of Tokat, the a* value was found to be 7.31.^32^ The above-mentioned literature results regarding the a* value, which is an important criterion in measuring the color of the service tree fruit peel, showed similarities with the a* value findings obtained in this study.

Differences in the b* value in color measurements of service tree genotypes were found to be statistically significant (*p* ≤ 0.05). While determining the fruit color, the *+b* value indicates that the color is yellow in the fruits, and the −*b* value indicates that the color is blue. In the measurement of the samples taken, the 14MR08 genotype had the highest b* value at 9.10, while the 14MR03 genotype had the lowest b* value at 5.04. The 14MR08 genotype was followed by the 14MR02 genotype with a 8.45 b* value and the 14MR010 genotype with a 8.40 b* value, respectively. The 14MR03 genotype was followed by the 14MR07 genotype with a b* value of 5.41 and the 14MR06 genotype with a b* value of 6.54, respectively (Table 3). In a study in which the skin color of service tree fruits was measured in the province of Tokat, the b* value was found to be 13.03.^32^ According to this literature result, which was determined regarding the b* value used in the measurement of the service tree fruit skin color, the b* value determined in this study was observed to be partially lower. It is thought that this partial difference in the b* value may be caused by genotype, geographical location, ecological factors, soil characteristics, and years.

Chroma refers to the intensity (saturation) of the color. In color value measurements, the differences of service tree genotypes in terms of chroma values were found to be statistically significant (*p* ≤ 0.05). While the 14MR09 genotype had the highest (12.87) chroma value in color measurements, the 14MR03 genotype had the lowest (9.08) chroma value. The 14MR09 genotype was followed by 14MR08 (12.29) and 14MR10 (12.25) genotypes, respectively. The 14MR03 genotype was followed by the 14MR07 (9.80) genotype (Table 3). In a study in which the skin color of service tree fruits was measured in the province of Tokat, the chroma value was found to be 15.^32^ This literature result regarding the chroma value was similar to the chroma value findings obtained in this study.

It is the distance from the vertical axis of the point in the hue color space that indicates the intensity of the color. The differences of service tree genotypes in terms of hue value in color measurements were found to be statistically significant (*p* ≤ 0.01). In the color measurements of the samples, the highest hue value (49.07) was detected in the 14MR04 genotype, and the lowest (31.24) hue value was determined in the 14MR07 genotype. 14MR04 genotype was followed by 14MR08 (47.76) and 14MR02 (46.98) genotypes, respectively (Table 3). In a study carried out to measure the fruit skin color of the service tree fruit, the criterion hue value was found to be between 1.29 and 1.30.^38^ In a study in which the skin color of service tree fruits was measured in the province of Tokat, the hue value was found to be 60.54.^32^ When the results of the literature and this study were compared with the results of the above-mentioned literature regarding the hue value used in the measurement of skin color in service tree fruits, it was observed that the highest hue value determined in this study was partially higher or lower than the results of the examined literature. Accordingly, it is thought that these differences in hue values may be caused by genotype, geographical location, ecological factors, soil characteristics, and years.

When the data were examined in terms of fruit astringency, the astringency value of service tree fruits was evaluated as 1: not acrid, 2: slightly acrid, 3: moderately acrid, and 4: astringent. In terms of astringency values of fruits, seven of the genotypes (14MR01, 14MR02, 14MR03, 14MR04, 14MR05, 14MR07, and 14MR10) in the study were determined as “not acrid”, and three (14MR06, 14MR08, and 14MR09) were determined as “slightly acrid” (Table 4). Accordingly, it is thought that the astringency value results determined in relation to the genotypes of service tree fruits in this study may be useful in the formation of the literature on service tree fruits.

**Table 4:** Fruit Astringency, Fruit Flavor, Fruit Juice Color, Fruit Flesh Color, and Fruit Taste Characteristics of Fruits Belonging to Service Tree Genotypes

genotypes	fruit astringency	fruit flavor	fruit juice color	fruit flesh color	fruit taste
14MR01	not	good	medium	dark brown	moderate
14MR02	not	good	dark	dark brown	moderate
14MR03	not	good	medium	dark brown	moderate
14MR04	not	good	medium	dark brown	good
14MR05	not	good	medium	dark brown	moderate
14MR06	slightly	good	dark	brown	moderate
14MR07	not	moderate	dark	brown	good
14MR08	slightly	moderate	medium	brown	moderate
14MR09	slightly	moderate	dark	brown	moderate
14MR10	not	moderate	light	dark brown	good

When the data were examined in terms of fruit aroma, the aroma value of service tree fruits was evaluated in the categories of 1: good, 2: medium, and 3: bad. In terms of flavor values of fruits, six of the genotypes (14MR01, 14MR02, 14MR03, 14MR04, 14MR05, and 14MR06) detected in the study were determined as “good”, and four (14MR07, 14MR08, 14MR09, and 14MR10) were determined as “moderate” (Table 4). Accordingly, it is thought that the aroma value results determined in relation to the genotypes of service tree fruits determined in this study may be useful in the formation of the literature on service tree fruits. When the data were examined in terms of juice color, the juice color of service tree genotypes was evaluated in the categories of “1: light brown”, “2: medium dark brown”, and “3: dark brown”. In terms of juice color, four of the genotypes (14MR01, 14MR03, 14MR04, and 14MR05) detected in the study were “medium dark brown”, four (14MR02, 14MR06, 14MR07, and 14MR09) were “dark brown”, and only one (14MR10) was determined as “light brown” (Table 4). Accordingly, it is thought that the fruit juice color results determined in relation to the genotypes of service tree fruits in this study may be useful in the formation of the literature on service tree fruits. When the data were examined in terms of fruit flesh color, the fruit flesh color of service tree genotypes was evaluated in the categories of “1: light brown”, “2: brown”, and “3: dark brown”. In terms of fruit flesh color, six of the genotypes (14MR01, 14MR02, 14MR03, 14MR04, 14MR05, and 14MR10) detected in the study were determined as “dark brown” and four (14MR06, 14MR07, 14MR08, and 14MR09) as “brown” (Table 4). Accordingly, it is thought that the fruit flesh color results determined in relation to the genotypes of service tree fruits in this study may be useful in the formation of the literature on service tree fruits. When the data were examined in terms of fruit taste, in this study, genotypes were evaluated in the categories of 1: poor, 2: medium, 3: good, and 4: very good according to fruit taste. In terms of fruit taste, seven of the genotypes (14MR01, 14MR02, 14MR03, 14MR05, 14MR06, 14MR08, and 14MR09) detected in the study were determined as “moderate”, and three (14MR04, 14MR07, and 14MR10) were determined as “good” (Table 4). Accordingly, it is thought that the fruit taste results determined in relation to the genotypes of service tree fruits in this study may be useful in the formation of the literature on service tree fruits.

### Phenolic Compound Content of Fruits

Tables 5 and 6 show phenolic compounds of service tree genotypes. The genotypes exhibited statistically significant differences between each other at the *p* ≤ 0.05 level. Service tree genotypes mostly included chlorogenic acid, ferulic acid, and rutin, while vanillic acid and *o*-coumaric acid were found to have the lowest value. The amount of gallic acid is 5.72 mg/100 kg (14MR04)–16.04 mg/100 kg (14MR05), the amount of catechin is 1.61 mg/100 kg (14MR02)–12.68 mg/100 kg (14MR07), the amount of caffeic acid is 2.15 mg/100 g (14MR02)–15.33 mg/100 kg (14MR04), the amount of vanillic acid is 0.67 mg/100 kg (14MR10)–2.51 mg/100 kg (14MR05), the amount of *p*-coumaric acid is 1.53 mg/100 kg (14MR01)–17.89 mg/100 kg (14MR05), the amount of chlorogenic acid is 29.18 mg/100 kg (14MR04)–48.49 mg/100 kg (14MR10), the amount of *o*-coumaric acid is 1.66 mg/100 kg (14MR02)–9.13 mg/100 kg (14MR03), the amount of ferulic acid is 5.06 mg/100 kg (14MR02)–36.93 mg/100 kg (14MR10), the amount of rutin is 7.90 mg/100 kg (14MR10)–36.98 mg/100 kg (14MR05), and the amount of quercetin is ranged between 4.09 mg/100 kg (14MR04) and 15.72 mg/100 kg (14MR01) (Tables 5 and 6). In a study investigating the specific phenolic compounds found in service tree fruits, vanillic acid was found to be the phenolic compound observed in the lowest amount.^40^ In another study, in which dominant phenolic compounds were detected in service tree fruits, it was noted that ferulic acid was among the dominant compounds.^41^ In a study in which dominant phenolic compounds were detected in service tree fruits, it was reported that chlorogenic acid was the dominant phenolic compound.^42^ Previously, rutin and chlorogenic acids were found to be dominant phenolic compounds in service tree fruits.^43^ Forino et al.^44^ found that chlorogenic acid is abundant in service tree fruits.

**Table 5:** Phenolic Compounds of Service Tree Fruits of Genotypes (mg/kg fw)

genotypes	gallic acid	catechin	chlorogenic acid	caffeic acid	vanillic acid
14MR01	15.55 ± 0.01ab^a^	2.97 ± 0.01g	41.75 ± 0.02e	5.12 ± 0.02h	2.14 ± 0.01b
14MR02	13.61 ± 0.03abc	1.61 ± 0.02i	46.32 ± 0.01b	2.15 ± 0.02i	2.10 ± 0.02b
14MR03	8.08 ± 0.01bc	8.98 ± 0.01d	42.16 ± 0.01d	10.67 ± 0.02c	0.87 ± 0.02g
14MR04	5.72 ± 4.48c	9.63 ± 0.01c	29.18 ± 0.02j	15.33 ± 0.03a	1.91 ± 0.02c
14MR05	16.04 ± 0.01a	6.44 ± 0.02e	37.21 ± 0.02g	9.14 ± 0.02d	2.51 ± 0.01a
14MR06	15.37 ± 0.02ab	1.85 ± 0.02h	29.50 ± 0.02i	6.22 ± 0.01g	1.75 ± 0.02d
14MR07	11.36 ± 0.01abc	12.68 ± 0.03a	33.93 ± 0.02h	12.22 ± 0.03b	1.62 ± 0.01e
14MR08	12.14 ± 0.02abc	10.11 ± 0.02b	39.39 ± 0.02f	7.13 ± 0.03f	0.98 ± 0.01f
14MR09	12.65 ± 0.02abc	4.14 ± 0.02f	45.96 ± 0.01c	6.21 ± 0.02g	1.81 ± 0.02cd
14MR10	15.63 ± 0.02ab	6.46 ± 0.02e	48.49 ± 0.03a	8.15 ± 0.02e	0.67 ± 0.01h

a The difference between the means with the same letter in the same column is significant at the *p* < 0.05 level.

**Table 6:** Continuation of Table 5 (mg/kg fw)

genotypes	*p*-coumaric acid	ferulic acid	rutin	*o*-coumaric acid	quercetin
14MR01	1.53 ± 0.01I^a^	6.80 ± 0.02h	11.98 ± 0.01g	2.02 ± 0.01h	15.72 ± 0.02a
14MR02	5.25 ± 0.02f	5.06 ± 0.01j	12.65 ± 0.02f	1.66 ± 0.01i	5.95 ± 0.02h
14MR03	7.77 ± 0.01b	13.65 ± 0.00f	9.81 ± 0.01h	9.13 ± 0.02a	7.89 ± 0.02d
14MR04	6.04 ± 0.01d	5.22 ± 0.01i	15.66 ± 0.01d	3.66 ± 0.03e	4.09 ± 0.02j
14MR05	17.89 ± 0.01a	27.33 ± 0.00c	36.98 ± 0.01a	2.61 ± 0.01f	11.14 ± 0.02b
14MR06	5.88 ± 0.01e	27.03 ± 0.02d	17.66 ± 0.02c	3.67 ± 0.02e	6.84 ± 0.01g
14MR07	4.70 ± 0.01g	32.58 ± 0.01b	13.03 ± 0.02e	4.20 ± 0.02d	9.15 ± 0.02c
14MR08	2.64 ± 0.02h	7.11 ± 0.02g	17.70 ± 0.02c	2.16 ± 0.01g	7.56 ± 0.01e
14MR09	7.32 ± 0.01c	24.93 ± 0.02e	31.44 ± 0.01b	6.57 ± 0.01b	4.68 ± 0.02i
14MR10	5.17 ± 0.02f	36.93 ± 0.02a	7.90 ± 0.09i	6.42 ± 0.01c	6.95 ± 0.02f

a The difference between the means with the same letter in the same column is significant at the *p* < 0.05 level.

### Organic Acid Content of Fruits

Organic acids contribute to the taste of fruits and play an important role in their metabolic events of fruits.^45^ The organic acid/sugar ratio is not only important for taste formation but also determines the suitability of fruits for processing into juice. Table 7 indicates the organic acid and vitamin C content of service tree fruits. Results revealed that statistically significant (*p* ≤ 0.05) differences were evident among genotypes for organic acids and vitamin C content. The fruits of 14MR01 had the highest citric acid value as 12.16 g/kg fw, while the 14MR09 genotype showed the lowest value of 5.17 g/kg fw (Table 7). The genotypes 14MR04 (11.02 g/kg fw) and 14MR07 (10.03 g/kg fw) also had higher citric acid content after 14MR01.

**Table 7:** Organic Acids and Vitamin C Content of Service Tree Fruits^a^

genotypes	citric acid (g/kgfw)	tartaric acid (g/kgfw)	malic acid (g/kgfw)	succinic acid (g/kgfw)	fumaric acid (g/kgfw)	vitamin C (mg/100 gfw)
14MR01	12.16 ± 0.13a	1.24 ± 0.11c	30.51 ± 0.44abc	4.89 ± 0.06bc	4.23 ± 0.41b	82.44 ± 5.89ab
14MR02	9.72 ± 0.00c	2.06 ± 0.05b	26.71 ± 1.54bcd	6.73 ± 0.92ab	4.65 ± 0.08ab	95.83 ± 0.25a
14MR03	6.42 ± 0.07f	1.22 ± 0.13c	24.42 ± 1.41cde	6.44 ± 0.80bc	2.07 ± 0.03c	75.08 ± 6.84abc
14MR04	11.02 ± 0.22b	1.07 ± 0.09cd	30.89 ± 0.51ab	6.01 ± 0.17bc	1.75 ± 0.01cd	72.28 ± 9.07abc
14MR05	8.54 ± 0.05d	3.58 ± 0.09a	22.92 ± 0.77de	5.10 ± 0.13bc	1.40 ± 0.04cd	63.16 ± 0.61bcd
14MR06	6.85 ± 0.13ef	1.07 ± 0.06cd	15.56 ± 1.08fg	4.41 ± 0.35bc	1.00 ± 0.03d	46.16 ± 4.18d
14MR07	10.03 ± 0.10c	0.70 ± 0.03d	34.14 ± 1.42a	9.00 ± 0.49a	4.86 ± 0.12ab	85.88 ± 3.11ab
14MR08	6.44 ± 0.36f	1.04 ± 0.07cd	18.54 ± 1.03efg	4.01 ± 0.13c	1.02 ± 0.03d	50.53 ± 1.61cd
14MR09	5.17 ± 0.14g	1.29 ± 0.04c	13.92 ± 0.09g	5.62 ± 0.07bc	0.98 ± 0.00d	42.37 ± 0.54d
14MR10	7.51 ± 0.24e	1.09 ± 0.04cd	21.83 ± 1.80def	9.15 ± 0.40a	5.37 ± 0.04a	63.93 ± 2.47bcd

a The difference between the means with the same letter in the same column is significant at the *p* < 0.05 level.

The highest tartaric acid was observed in the 14MR05 genotype as 3.58 g/kg fw, followed by the 14MR02 (2.06 g/kg fw). Overall, the 14MR07 genotype had the lowest tartaric acid value (0.70 g/kg fw). Malic acid and succinic acid contents were found between 13.92 g/kg fw (14MR09)–34.14 g/kg fw (14MR07) and 4.01 g/kg fw (14MR08)–9.15 g/kg fw (14MR10), respectively. Fumaric acid was the lowest in 14MR09 (0.98 mg/kg), while it was the highest in 14MR010 (5.37 mg/kg). In this study, no literature study was found on the content of malic acid, which was determined as the dominant organic acid in *S. domestica*. Accordingly, it is thought that the findings related to the malic acid content of *S. domestica* in this study may be beneficial in the formation of the literature on service tree fruits.

Service tree genotypes present different amounts of vitamin C, and the differences in vitamin C were found to be statistically significant (*p* ≤ 0.05).

Vitamin C contents were in the following range of descending order: 14MR02 (95.83 mg/100 g) > 14MR07 (85.88 mg/100 g) > 14MR01 (82.44 mg/100 g) > 14MR03 (75.08 mg/100 g) > 14MR04 (72.28 mg/100 g) > 14MR10 (63.93 mg/100 g) > 14MR05 (63.16 mg/100 g) > 14MR08 (50.53 mg/100 g) > 14MR06 (46.16 mg/100 g) > 14MR09 (42.37 mg/100 g) (Table 7). In a study investigating the ascorbic acid content in service tree fruits, the ascorbic acid content was determined as 290 mg/100 g.^46^ In another study in which ascorbic acid was determined in service tree fruits, the amount of ascorbic acid was expressed as 22.65 mg/100 g.^47^ When compared with the results of the above literature studies in terms of vitamin C content, it was observed that the findings obtained in this study were significantly higher or lower than the others. Accordingly, it is thought that these differences in ascorbic acid may be caused by differences in environmental, genetic, and cultural factors.^48−55^

### PCA of Morphological–Physicochemical and Biochemical Properties

**Figure 1:**
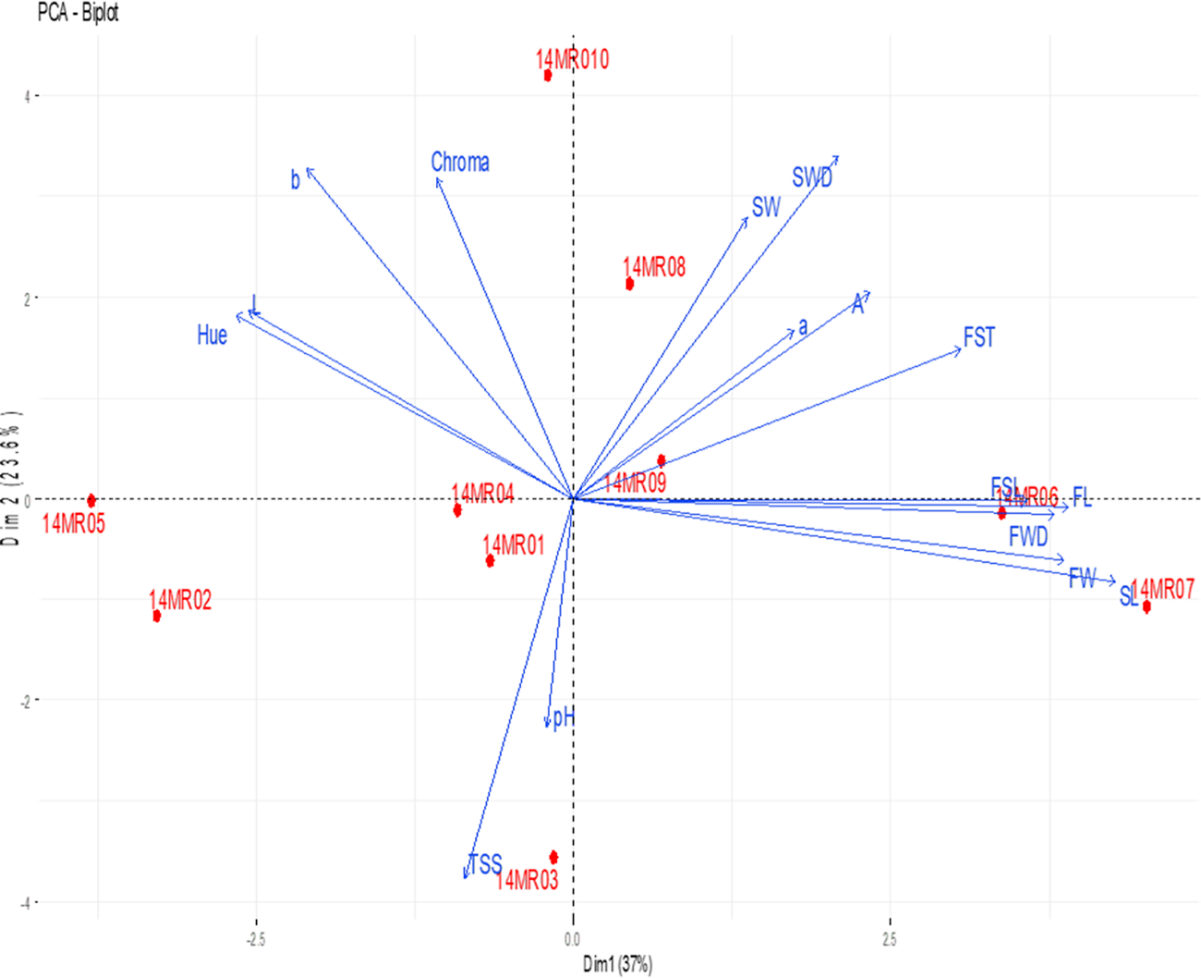
Correlation between agro-morphological and some biochemical properties of fruits belonging to genotypes. FSL: fruit stalk length, FST: fruit stalk thickness, FW: fruit weight, FWD: fruit width, FL: fruit length, SW: seed weight, SWD: seed width, SL: seed length, L: L* value, a: a* value, b: b* value, Chroma: chroma value, Hue: hue value, pH: juice pH, TSS: total soluble solids, and TA: titratable acidity.

The basic coordinate plane distributions for defining the correlation between some morphological and physicochemical properties of fruits belonging to service tree genotypes by PCA are given in Figure 1.

As seen in Figure 1, the first and second principal component axes account for 37 and 23.6% of the total variation, respectively. It was determined that the 14MR010 genotype was different from other genotypes in terms of morphological and physicochemical characteristics among the genotypes. In the two-dimensional graph, it is seen that some genotypes are located far from other genotypes. This result shows that some genotypes differ from other genotypes in terms of morphological and physicochemical characteristics. It has been determined that a*, L*, b*, chroma, and hue values of the parameters defined by PCA are parallel to each other. Similarly, it was determined that fruit weight and seed weight and fruit dimensions (width and length) and seed dimensions (width and length) were parallel to each other. Likewise, fruit stem length and fruit stem thickness showed parallelism with each other. In addition, it was determined that the pH of the juice and the SSC of the juice were parallel to each other, and the TA had a negative relationship.

**Figure 2:**
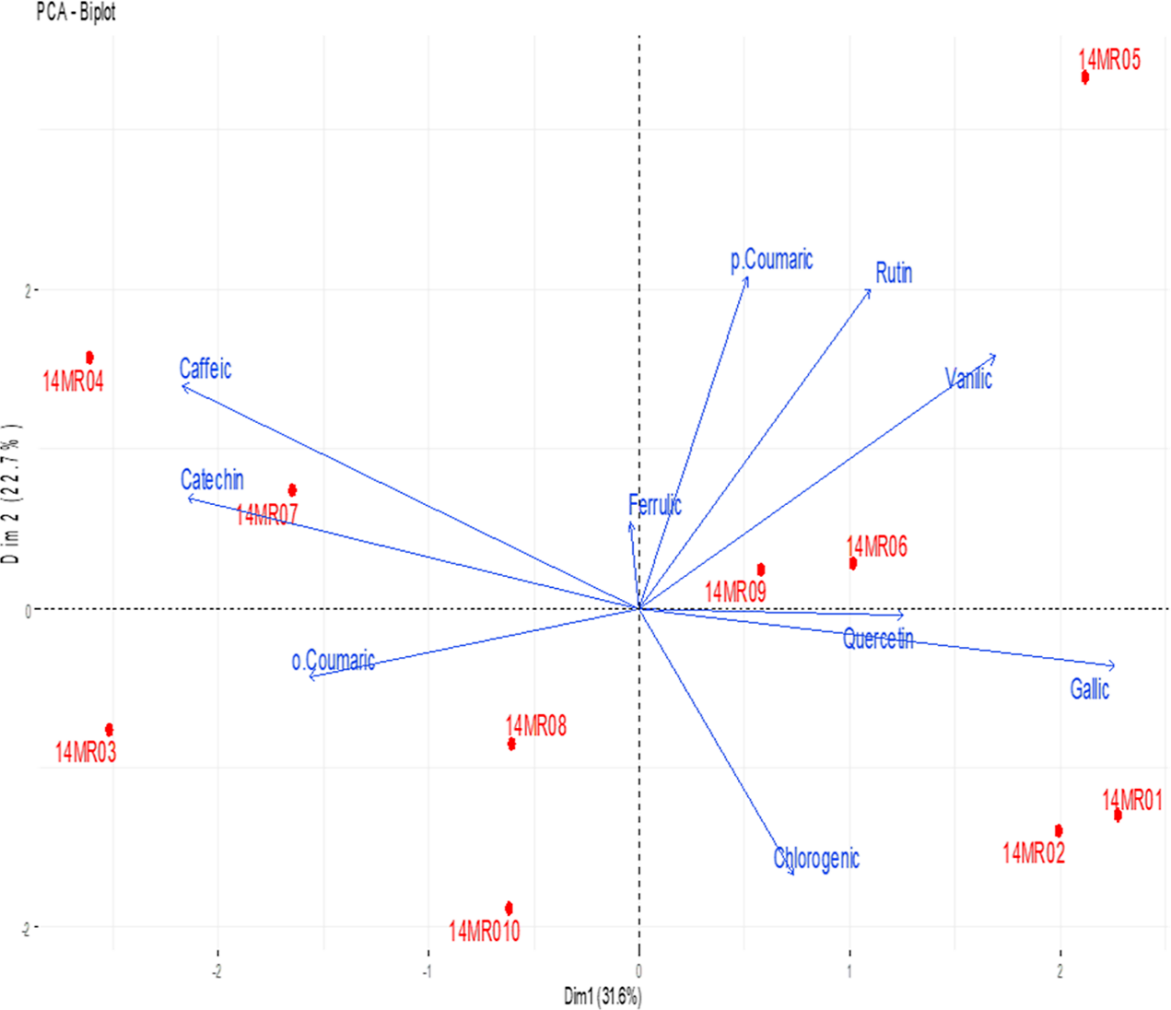
Correlation between phenolic compounds of fruits belonging to genotypes. Gallic: gallic acid, Catechin: catechin, Caffeic: caffeic acid, Vanillic: vanillic acid, *p*-Coumaric: *p*-coumaric acid, Chlorogenic: chlorogenic acid, *o*-Coumaric: *o*-coumaric acid, Ferulic: ferulic acid, Rutin: rutin, and Quercetin: quercetin.

The coordinate plane distributions for defining the correlation between phenolic compound properties of fruits belonging to service tree genotypes by PCA are given in Figure 2. When the results are examined, it is seen that the total variation is explained significantly by the first two principal component axes with a total value of 54.3% (31.6% first and 22.7% second principal component axes). These axes were found to be important in the evaluation of the analysis. It was determined that the 14MR05 genotype was different from other genotypes in terms of phenolic compound properties among genotypes. The graph also indicates that some genotypes are far from the others in terms of phenolic compound properties. Among the parameters defined by PCA, *p*-coumaric acid, rutin, vanillic acid, quercetin, and gallic acids were found to show parallelism with each other. Similarly, it was determined that caffeic acid, catechin, and *o*-coumaric acids showed parallelism with each other. In addition, ferulic acid and chlorogenic acid were found to have an opposite relationship with each other.

**Figure 3:**
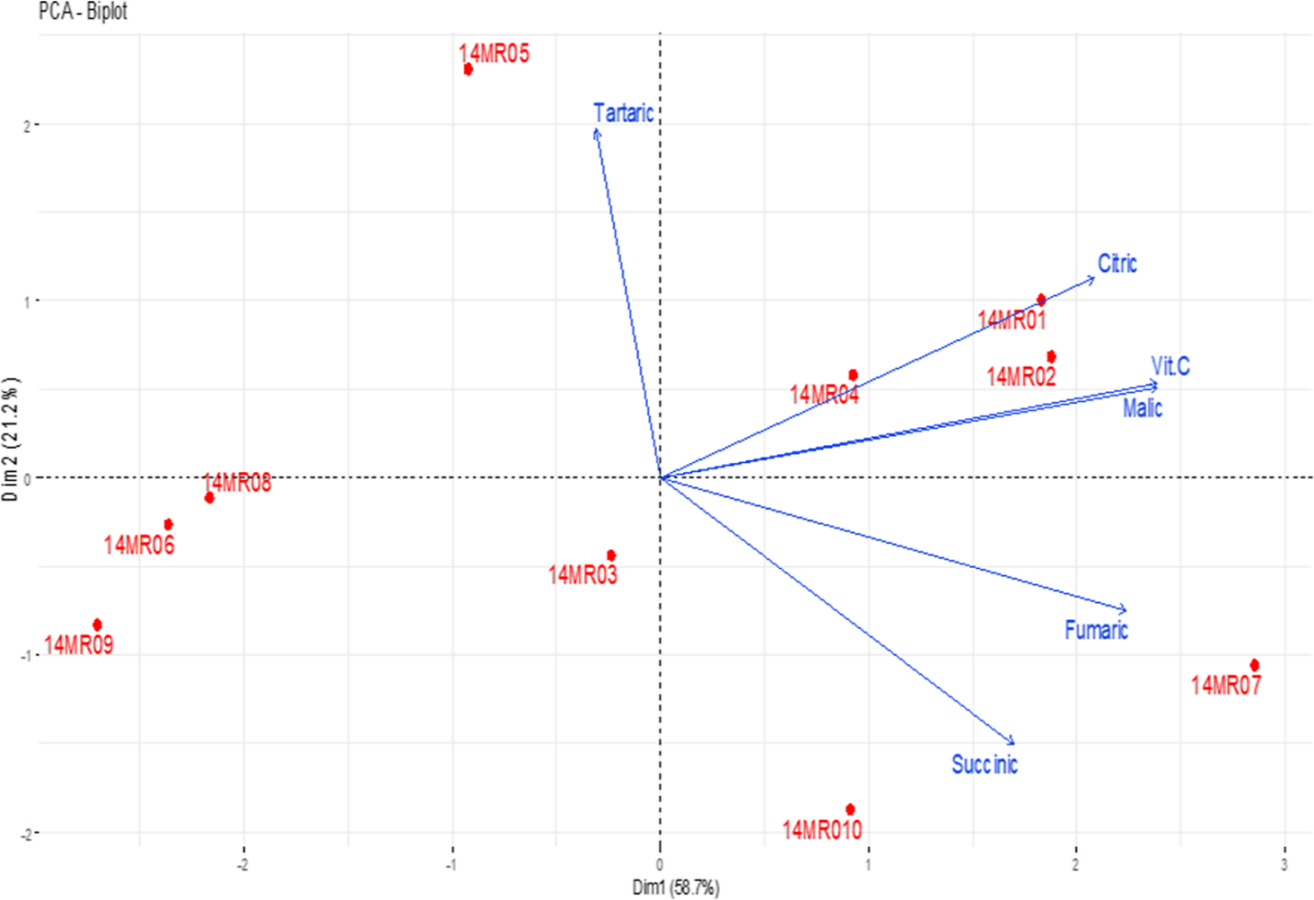
Correlation between organic acid and vitamin C contents of fruits belonging to genotypes. Citric: citric acid, Tartaric: tartaric acid, Malic: malic acid, Succinic: succinic acid, Fumaric: fumaric acid, and Vit.C: vitamin C.

The basic coordinate plane distributions for defining the correlation between organic acids and vitamin C properties of fruits belonging to service tree genotypes by PCA are given in Figure 3. When the results are examined, it is seen that the total variation is explained significantly by the first two principal component axes with a value of 79.9%. The first principal component axis accounts for 58.7% of the total variation, and the second principal component axis covers 21.2% of the total variation. These axes were found to be important in the evaluation of the analysis. Among the genotypes, it was determined that the 14MR05 genotype was different from the other genotypes in terms of organic acids and vitamin C properties. In the two-dimensional graph, it is seen that some genotypes are located far from other genotypes. This result shows that some genotypes are different from other genotypes in terms of organic acids and vitamin C properties. It has been determined that the values of citric acid, malic acid, fumaric acid, succinic acid, and vitamin C, which are among the parameters defined by PCA, show parallelism with each other, and tartaric acid has a negative relationship with these values.

## Conclusions

In this study, morphological, physicochemical, and biochemical properties in fruits of 10 service tree (*S. domestica*) genotypes grown in Bolu province were investigated. In the examinations made in terms of morphological and physicochemical contents, 14MR07, 14MR06, 14MR04, and 14MR01 genotypes were found to be promising in terms of fruit weight. The fruit sizes determined in the study were observed in accordance with the fruit weight. SSC content in fruits is one of the basic criteria that is important in determining the maturity time and therefore can directly affect consumption. In this study, it was determined that the 14MR03, 14MR01, and 14MR04 genotypes contain at least 20% SSC and are superior to other genotypes in these aspects. In the examinations made in terms of biochemical contents, it was determined that chlorogenic acid, ferulic acid, and rutin compounds had higher rates than other phenolic compounds. Malic acid was dominant for all genotypes. The 14MR02 genotype had a distinctly higher vitamin C content. The genotypes with distinct horticultural characteristics and higher human health-promoting content could be consider as breeding material in the production of functional food as well.*C*
